# Potential Cost-Effectiveness of a New Infant Tuberculosis Vaccine in South Africa - Implications for Clinical Trials: A Decision Analysis

**DOI:** 10.1371/journal.pone.0083526

**Published:** 2014-01-15

**Authors:** Jared B. Ditkowsky, Kevin Schwartzman

**Affiliations:** 1 Respiratory Epidemiology and Clinical Research Unit, Montreal Chest Institute, Montreal, Quebec, Canada; 2 Respiratory Division, Faculty of Medicine, McGill University, Montreal, Quebec, Canada; Archivel Farma; Fundació Institut d'Investigació en Ciències de la Salut Germans Trias i Pujol. Universitat Autònoma de Barcelona. CIBERES, Spain

## Abstract

Novel tuberculosis vaccines are in varying stages of pre-clinical and clinical development. This study seeks to estimate the potential cost-effectiveness of a BCG booster vaccine, while accounting for costs of large-scale clinical trials, using the MVA85A vaccine as a case study for estimating potential costs. We conducted a decision analysis from the societal perspective, using a 10-year time frame and a 3% discount rate. We predicted active tuberculosis cases and tuberculosis-related costs for a hypothetical cohort of 960,763 South African newborns (total born in 2009). We compared neonatal vaccination with bacille Calmette-Guérin alone to vaccination with bacille Calmette-Guérin plus a booster vaccine at 4 months. We considered booster efficacy estimates ranging from 40% to 70%, relative to bacille Calmette-Guérin alone. We accounted for the costs of Phase III clinical trials. The booster vaccine was assumed to prevent progression to active tuberculosis after childhood infection, with protection decreasing linearly over 10 years. Trial costs were prorated to South Africa's global share of bacille Calmette-Guérin vaccination. Vaccination with bacille Calmette-Guérin alone resulted in estimated tuberculosis-related costs of $89.91 million 2012 USD, and 13,610 tuberculosis cases in the birth cohort, over the 10 years. Addition of the booster resulted in estimated cost savings of $7.69–$16.68 million USD, and 2,800–4,160 cases averted, for assumed efficacy values ranging from 40%–70%. A booster tuberculosis vaccine in infancy may result in net societal cost savings as well as fewer active tuberculosis cases, even if efficacy is relatively modest and large scale Phase III studies are required.

## Introduction

Nearly one-third of the world's population is infected with *Mycobacterium tuberculosis* (*M. tuberculosis*), with an estimated 9.4 million incident cases and 1.3 million deaths in 2009 [1], [2]. Progress in reducing morbidity and mortality has been severely hampered by several challenges, including HIV co-infection, antibiotic resistance, and limited diagnostic and treatment capacity in many high-burden settings. As a consequence, tuberculosis (TB) control strategies are evolving to address novel diagnostic tools, treatment regimens for multi-drug resistance, TB-HIV program integration, and other potential solutions. For TB control in the longer term, modeling studies have underlined the importance of enhanced diagnostic capacity, expanded treatment of latent TB infection, new anti-tuberculosis drugs, and improved TB vaccines [1]. In principle, an effective TB vaccine can circumvent some of the challenges posed by drug resistance, treatment adherence, and potentially HIV-TB [1].

The bacille Calmette-Guérin (BCG) vaccine is the only vaccine currently licensed for TB prevention. While it is considered modestly efficacious in preventing tuberculosis meningitis and disseminated TB in children, estimates of its true efficacy vary widely [3]. Furthermore, it appears to provide limited or no protection against adult pulmonary TB, so that it cannot directly reduce transmission [4], [5]. Because of these gaps, the last decade has witnessed substantial interest and investment in TB vaccine development. Select candidates, including the Aeras 402/Crucell Ad35 and MVA85A vaccines have entered Phase IIB clinical trials for safety and efficacy, based on promising Phase I safety and immunogenicity data. The MVA85A vaccine has recently undergone evaluation in BCG-vaccinated infants in South Africa, and is now being studied in HIV-infected adults in Senegal and South Africa [6], [7], [8], in trials conducted by the Oxford-Emergent Tuberculosis Consortium (OETC).

MVA85A vaccination of persons with previous exposure to *M. tuberculosis* or BCG vaccination appears to result in increased immunogenicity, compared to MVA85A vaccination of BCG/*M. tuberculosis*-naïve individuals [9], [10]. However, a phase IIB randomized, controlled trial among 2,797 South African infants demonstrated no additional efficacy beyond BCG alone [11]. Hence while immunological data for some new vaccines are promising, the substantial resources required for clinical development and rollout must be evaluated in the context of the potential downstream health benefits and cost savings. This is particularly relevant for funders and decision makers, who must consider further investments in vaccine development, versus investments in other promising approaches to TB control. Using a potential infant booster vaccination program as a case study, we examined the balance between vaccine development and administration costs, including those of further clinical trials, and later gains in TB morbidity, mortality, and the related cost savings.

## Methods

**Figure 1:**
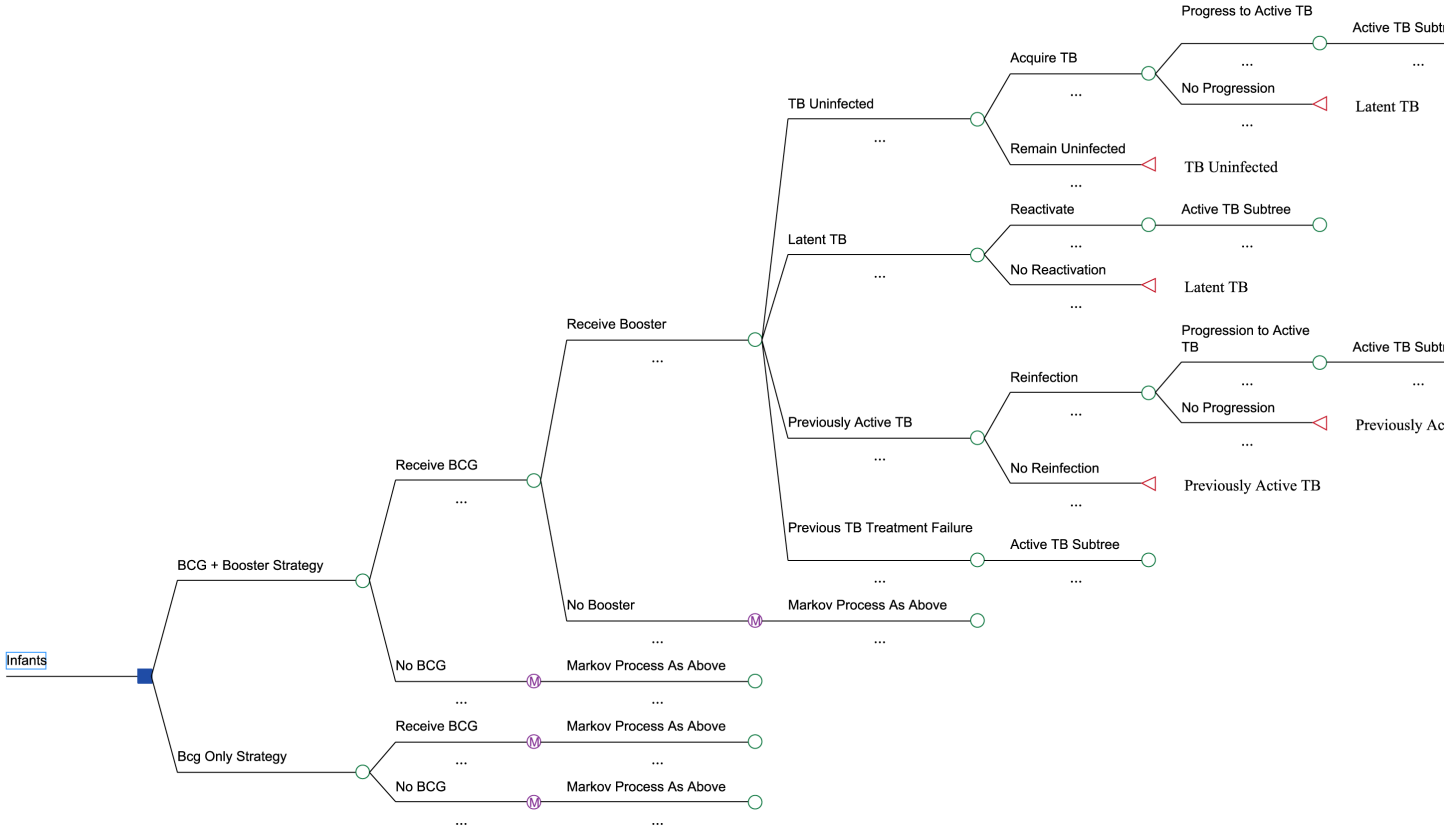
Markov process used to estimate vaccination rates, acquisition of latent TB infection and active disease.

**Figure 2:**
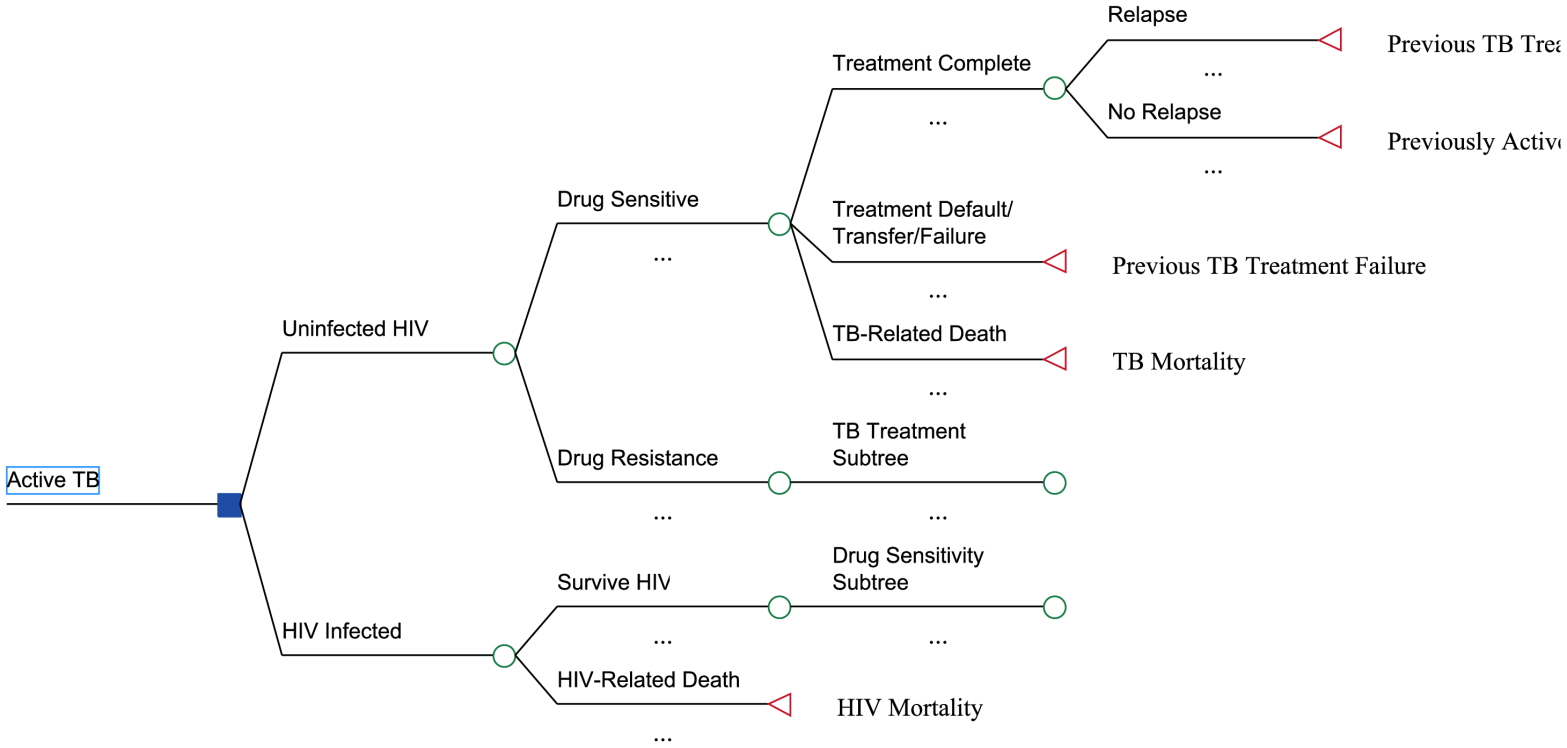
Sample subtree showing potential drug resistance and treatment outcomes after diagnosis of active TB disease.

We developed a Markov model, using TreeAge ProSuite 2009 (TreeAge Software, Williamstown, MA.) We compared two scenarios: 1) current neonatal BCG vaccination and DOTS coverage, without booster vaccination, and 2) current neonatal BCG vaccination and DOTS coverage, plus a new infant booster vaccine administered at age 4 months. (Figures 1–2). The model was calibrated to characteristics of the South African population, and examined a cohort of 960, 763 newborns entering the population (total born in 2009). We predicted active TB cases, TB deaths and related costs. The analysis was conducted from the South African societal perspective. We used a 10-year time frame, and a 3% discount rate [12].

Population and TB control parameters are listed in Table 1; these were assumed to remain constant over the 10-year simulation period. BCG vaccination was assumed to provide an initial 50% reduction in the rate of primary progression to TB disease, decreasing linearly to zero protection at the end of the 10 years [4], [12].

**Table 1:** Epidemiologic and Population Characteristics for South Africa.

Population Characteristics	Value	Source
Population (2009)	49,320,500	[32]
Birth rate per individual (2009)	0.01948	[33]
Percentage of global BCG vaccine coverage	0.08%	Calculated [26], [33]–[34]
Percentage of target population vaccinated with BCG (2010)	90%	[34]
Gross National Income per capita (2012 USD)	5,760	[35]
Life expectancy at birth in years (2011)	49.315	[36]
All cause mortality	Age Dependent/Interpolated	[13]
TB annual risk of infection	4.3%	[37]
HIV prevalence in newborns (2009)	5.45%	[38]
HIV annual risk of infection for infants	0	Assumption
Probability eligible child is screened for LTBI*	50%	Assumption
Probability LTBI treatment is provided when indicated	34%	[19]
LTBI treatment efficacy	78%	[20]
LTBI treatment completion	20%	[20]
DOTS coverage	Universal	[39]
DOTS case detection rate	63.2%	Calculated [40]
Initial drug resistance		
Single drug resistance	6.60%	[41]
Multi drug resistance† (2009)	1.80%	[40]
DOTS new child TB case treatment outcome		
Cure/complete	72.0%	[42]
Default/transfer/not evaluated	16.6%	[42]
Die	11.3%	[42]
Fail	0.1%	[42]

Assumes only HIV-infected children and those with family contacts with active TB are potentially tested and treated for LTBI.

†Defined as resistance to isoniazid and rifampin, with or without other drug resistance.

We assumed 90% BCG coverage of newborns [13], and that among BCG-vaccinated infants, 90% would receive the booster if offered. Booster vaccine protection waned linearly to zero over its 10-year duration of action. In the base case analysis, the booster vaccine was first assumed to confer 60% protection over BCG alone [14]. We then varied the assumed efficacy from 40% to 70% over BCG alone. Neither vaccine was considered to provide any protection to children with clinical AIDS.

Infants could acquire TB infection at any stage of the simulation. After acquiring TB infection, they could progress immediately to active TB disease, or remain with latent infection. Initial infection with HIV led to an “early HIV” status. Progression to clinical AIDS could occur at any stage after initial infection. Based on South African data, we assumed that anti-retroviral therapy (ART) was offered to 58% of infected infants, and was associated with a 75% decrease in HIV-related annual mortality for those with early HIV, and a 9.8% decrease for those who progressed to clinical AIDS [15], [16], [17].

In addition, we assumed that isoniazid prophylaxis was provided only to children under 5 years of age who had HIV, and/or family contacts with active TB [18]. We assumed that 50% of eligible infants with latent tuberculosis would be screened. Of those screened, 34% would be provided treatment [19]. Treatment had an efficacy of 78%, with a 20% probability of treatment completion [20]. Incomplete treatment was assumed to be ineffective. Other clinical parameters are described in Table 2.

**Table 2:** Disease Variables.

Pathogenetic Variables	Value	Source
**HIV Infection**		
Risk of progression to AIDS at or near birth if HIV-infected	13%	Calculated [38], [43]
Annual risk of progression - asymptomatic HIV to AIDS	7%	[44]
Annual risk of death first year – asymptomatic	39%	[45]
Annual risk of death in first year - clinical AIDS	70%	[45]
Annual risk of death - early HIV	Age Dependent/Interpolated	[45]–[47]
Annual risk of death - clinical AIDS	Age Dependent/Interpolated	[45]–[47]
Probability of receiving ART if HIV-infected	58%	[15]
Protection against progression to AIDS with ART	61%	[17]
Annual reduction in mortality with ART – asymptomatic	75%	[15]
Annual reduction in mortality with ART - AIDS	9.8%	[17]
**Risk of developing Active TB Disease**		
**HIV Uninfected**		
Within 2 years of new TB infection	5%	[47]–[48]
Within 2 years of re-infection after cured TB disease	1%	[49]–[50]
Late re-activation from longstanding latent TB	1%/yr	[51]–[52]
**Early HIV**		
Within 2 years of new TB infection	33%	Extrapolated
Within 2 years of re-infection after cured TB disease	33%	Assumption
Late re-activation from longstanding latent TB	3.4%/yr	[53]–[55]
**Clinical AIDS**		
Within 2 years of new TB infection	100%	[56]–[60]
Within 2 years of re-infection after cured TB disease	100%	Assumption
Late re-activation from longstanding latent TB*	33%/yr	[55]
**Untreated TB Outcomes (HIV-negative)**		
Spontaneous resolution: **15% at age 1; 36% at age 10**	Age Dependent/Linear Inerpolation	[61]
Relapse after spontaneous resolution	2.5%/yr	[62]–[63]
Mortality rate for untreated TB	33% after 1 yr; 50% after 2 yrs	[64]
**Untreated TB Outcomes (HIV-positive)**		
Spontaneous resolution	0%	Assumption
Mortality rate within 2 years	100%	Assumption
**Treated TB outcomes (HIV-negative)**		
Relapse after cure (total over next 2 years)	3.00%	[65]–[69]
Cure rate if default (single drug resistant or drug sensitive)†	62.40%	[68]–[72]
**Effect of Drug Sensitivity on Treatment Outcomes**		
Relative risk of treatment failure- single drug resistant	2	[73]
Relative risk of treatment failure- multi-drug resistant	10.5	[73]
Relative risk of death- single drug resistant	1	[73]
Relative risk of death - multi-drug resistant	4	[73]
**Multi-Drug Resistant TB Treatment Outcomes**		
Completed/Cured	62.4%	Calculated [42], [73]–[74]
Default/Failed/Transferred	26.8%	Calculated [42], [73]–[74]
Died	10.8%	Calculated [42], [73]–[74]
**Treated TB Outcomes (HIV-positive)**		
Relative risk of death during TB treatment with HIV infection	3.6	[42], [75]
Relapse after successful TB treatment (cured)	3.10%	[76]–[79]

Assumes risk of reactivation more than 2 years after TB infection is the same whether first infection or reinfection.

†Transfer considered as default.

Costs were expressed in 2012 US dollars. We included all TB-related health care costs, as well as family expenditures related to children's illness, and productivity losses by family members. South African gross national income (GNI) per capita was used to calculate income and productivity losses based on a 40-hour work week. Where possible, costs were obtained from previous cost-effectiveness analyses addressing TB treatment in South Africa [21], [22]. Drug costs reflected the Global Drug Facility price list, and South African treatment recommendations [23], [24]. DOTS program administrative costs were based on earlier evaluations of DOTS implementation in South Africa (Tables 3 and 4) [25].

**Table 3:** Direct and Indirect Costs per tuberculosis patient managed in South Africa.

Type of Cost	Mean	Source
	(cost: $US 2012)	
**Pre-Diagnosis**		
Number of medical visits	4	[21]
Lab Costs/Health Care System costs (3 AFB smears)	$19.45	[21], [55]
Patient out-of-pocket expenditures for visit	$10.05	[22]
Indirect - Lost income for family per visit*	$3.70	[21]
**Post-Diagnosis**		
Hospitalization- Number of hospital days	3	[1]
Direct - Health system costs for hospital day	$23.29	[22]
Patient out-of-pocket expenditures: hospital days	$11.55	[22]
Indirect - Lost income for family per hospital day*	$23.79	[80]
**Directly Observed Treatment**		
Number of visits	130	[21]
Direct - Health system costs for visit	$5.32	[21]
Drug costs (new case)	$15.10	[23]–[24]
Patient out-of-pocket expenditures for visits	$0.29	[21]
Indirect - Lost income for family per visit*	$2.29	[21]
**Follow-up**		
Number of visits	3	[21]
Direct-Health system costs for visit	$23.29	[21]
Patient out-of-pocket expenditures for visits	$0.39	[21]
Indirect - Lost income for family per visit*	$2.48	[21]
**Total Cost per TB patient managed**		
Direct - Health System	$871.94	[21]
Patient out-of-pocket and miscellaneous costs	$54.36	[21]
Indirect - Family lost income and miscellaneous costs	$416.14	[21]
Total - Health system and patient/family	$1,342.43	[21]

Based on gross national income per capita.

**Table 4:** Vaccine-Associated Costs.

Vaccine Costs	Unit Cost	Source
**Booster Vaccine**		
Research and Development	$0.34	*
Direct Production	$2.00	[81,*]
Distribution	$0.70	[26]
Administration	$0.50	[26]
Profit Margin	$4.00	*
Total Unit Cost†	$7.54	
**BCG Vaccine**		
Direct Production BCG	$0.93	[5], [26]
Distribution BCG	$0.70	[26]
Administration BCG	$0.50	[26]
Total Unit Cost	$2.13	

Cost scenarios gathered in part by interview with Oxford Emergent Tuberculosis Consortium.

Further research and development costs for a booster vaccine reflected estimated costs of Phase IIB and potential Phase III clinical trials of the MVA85A vaccine. Initial pre-clinical development and early phase clinical study costs were considered to be sunk i.e. already spent, as is also the case for other current leading vaccine candidates, so they were not included. Sample size and cost information was based on interviews with the OETC, which has not otherwise contributed to or reviewed this paper. The initial sample size calculation for a phase III trial involves a hypothesized efficacy of 60%, and is predicated on a lower limit of 30% vaccine efficacy for the 95% confidence interval. The estimated baseline risk of active TB is 1% per year. With these parameters, the target sample size is a total of 12,000 infants, with a total cost of $120 million, including $30 million in start-up/infrastructure costs, and $90 million for recruitment and follow-up ($7,500 per subject). Phase IIB trial costs, estimated at $30 million, plus an additional $10 million for infrastructure, were also added. [Of note, Phase I and II trials of the final commercial formulation of any vaccine will also be required and additional lot-to-lot Phase III consistency trials may be required depending upon the final design of the Phase III trial]. OETC reported that Aeras has previously publicly stated that the eventual target cost of vaccine production for use in the developing world is $2/dose. In fact, OETC anticipates that vaccine production costs would not reach this level without considerable economies of scale and purpose-built manufacturing facilities.

For the purposes of the present analysis, the total estimated Phase III trial cost was divided by the number of vaccine doses to be administered worldwide over 10 years after its introduction (based on 106.4 million BCG doses administered annually [26]), so as to attribute a share of the trial cost to every dose. This assumes that the trial cost would be recouped over the first 10 years of vaccine use. Phase III trial parameters are listed in Table 5. The Phase IIB trial costs (total $40 million) were also incorporated into the final research and development cost, and were attributed to each dose in the same way as Phase III costs. We examined alternate scenarios for true vaccine efficacy - while keeping 30% as the lower limit of the 95% confidence interval from any trial - and the resulting changes in sample size and cost. The target cost of vaccine production was initially set at $2/dose. In the base case analysis, we assumed a profit margin of $4/dose.

**Table 5:** Sample Size and Research Cost for Different Booster Vaccine Efficacy Values.

Relative Efficacy	Cumulative TB Incidence	Phase III	Research Cost
Over BCG Alone	with Combined Vaccination	Sample Size*	($millions US 2012)
	(2 years)		Phase IIb plus Phase III†
70%	0.6%	5,342	87.87
65%	0.7%	7,215	108.16
60%	0.8%	10,141	139.86
55%	0.9%	15,063	193.18
50%	1.0%	24,251	292.72
45%	1.1%	44,380	510.78
40%	1.2%	102,696	1,092.54

Length of follow-up 2 years.

assumed active TB risk = 2% in control arm;

lower limit confidence interval set to >30%;

Power 90%;

Significance level = .05.

†The estimated cost of Phase IIB (clinical trials plus start-up costs), added to the cost of Phase III trials to give a final research and development cost.

We conducted extensive sensitivity analyses for all assumed parameters, with epidemiologic parameters varied across published ranges. Sensitivity analyses included a multiway probabilistic analysis, with simultaneous variation of the parameters with the largest impact on predicted outcomes. We used a triangular distribution, where the base case value was most likely, and the low and high extremes of the distribution were half and double the base case assumption respectively. The probabilistic analysis consisted of 100 runs and 100,000 microsimulations.

## Results

### Base Case

**Figure 3:**
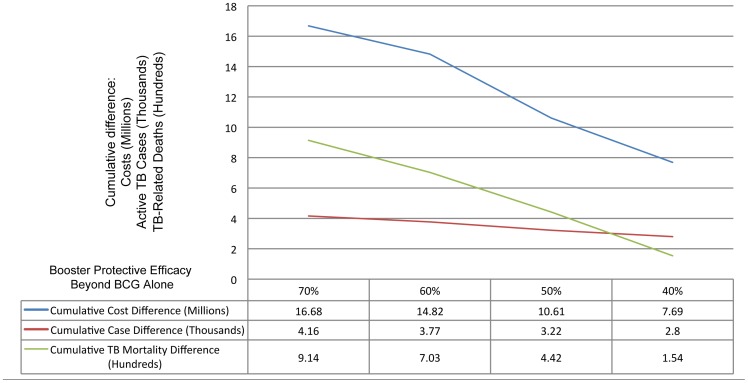
Total number of TB-cases, TB-related mortality and costs averted with different scenarios for booster efficacy.

For the birth cohort of 960,763 infants, we projected that the current BCG/DOTS strategy would cost $89.91 million over 10 years, with 13,607 active TB cases and 3,243 TB-related deaths over the same period. Assuming that the protective efficacy of the combined BCG-booster vaccination was 60% relative to BCG alone, we projected savings of $14.82 million, with 3,772 TB cases and 703 deaths averted. As expected, higher efficacy estimates resulted in greater cost savings as well as further improvements in morbidity and mortality. However, with an efficacy of 40% for the combined vaccinations, relative to BCG alone, there were still projected cost savings—even after accounting for the attendant increase in clinical trial sample size requirements (Table 6 and Figure 3).

**Table 6:** Predicted Outcomes with Varying Efficacy Values for BCG+Booster Vaccine.

Protection Increase relative to BCG Alone	Total Cost* BCG Alone	Total Cost* Booster	Difference Cost*	Cases Active TB† BCG Alone	Cases Active TB† Booster	Difference Active TB†	TB Mortality† BCG Alone	TB Mortality† Booster	Difference TB Mortality†
70%	89.91	73.23	16.68	13.61	9.45	4.16	3.24	2.33	.91
60%	89.91	75.09	14.82	13.61	9.84	3.77	3.24	2.54	.7
50%	89.91	79.30	10.61	13.61	10.39	3.22	3.24	2.80	.44
40%	89.91	82.22	7.69	13.61	10.81	2.8	3.24	3.09	.15

Cost expressed in $million US 2012.

†Expressed in thousands.

### Sensitivity Analyses

When parameters that directly affected vaccine costs were varied, the booster vaccination strategy remained cost saving in most cases. For example, when the per-dose cost of the booster vaccine was doubled, there were still cost savings of $6.08 million for the booster strategy. With the assumption that the duration of vaccine action was halved, to only 5 years, the booster strategy resulted in a net cost of $1.24 million. The threshold value for cost savings was ≥5.72 years of vaccine activity.

In most other one-way sensitivity analyses, the booster strategy remained cost-saving relative to BCG alone, in addition to reducing TB morbidity and mortality. With the base case assumptions, any profit margin less than $17.13 per vaccine dose will still result in net cost savings for South African society (Table 7).

**Table 7:** Sensitivity Analysis.

Varied Parameter	TB Cases Prevented by Addition of Booster	TB Deaths Prevented	Savings $million US 2012	Threshold
Base Case	3,772	703	14.82	
Cost Unit Booster Vaccine				
halved to $3.80	N/A	N/A	15.18	
doubled to $15.20	N/A	N/A	6.08	
Profit Margin				
halved to $2	N/A	N/A	15.82	
doubled to $8	N/A	N/A	8.96	
high end at $32	N/A	N/A	−10.74	$17.13/vaccine dose
Total Cost DOTS Visit				
halved to $4.04	N/A	N/A	11.17	
doubled to $16.16	N/A	N/A	17.86	
Protection Factor Post-TB Infection				
at Zero	3,702	703	16.41	
doubled to 40%	3,984	703	12.19	
TB annual risk of infection				
Low End 2.5%	1,158	525	4.83	
High End 8.14%	7,113	1,059	24.35	
Prevalence Rate HIV				
halved to .55%	3,129	865	11.94	
doubled to 2.18%	4,981	1056	17.68	
Probability Multi-Drug Resistance				
halved to 0.9%	3,574	748	14.20	
doubled to 3.6%	4,256	673	17.01	
Probability Single Drug Resistance				
halved to 3.3%	3,216	763	11.37	
doubled to 13.2%	5,732	629	19.62	
Discount Rate				
halved to 1.5%	5,216	847	20.79	
doubled to 6.0%	3,173	646	9.58	
Booster Mechanism - protection against initial infection	5,492	792	15.29	
Booster duration of action - halved, 5 yrs	694	417	−1.24	5.72 yrs duration of action
TB Prophylaxis				
0.25× probability, 0.663%	4,026	725	14.91	
5× probability, 13.26%	3,474	674	14.33	
ART, Protection from EHIV Progression				
halved to 30.5%	4,981	785	16.92	
High end, 90%	2,961	613	11.68	
Best Case Scenario*	6,038	4,854	38.56	
Worst Case Scenario†	941	137	−7.32	

Best Case Scenario.

**Halved**: Booster Vaccine Cost per Dose; Probability of TB Diagnosis; ART protection from EHIV progression.

**Doubled**: Probability of Drug-Resistant TB, HIV Prevalence at Birth; Cost per DOTS visit.

**BCG + Booster Vaccine Efficacy** = **85% relative to BCG alone.**

†Worst Case Scenario:

**Halved:** Probability of Drug-Resistant TB; HIV Prevalence at Birth; Cost per DOTS visit.

**Doubled**: Booster Vaccine Cost per Dose.

**ART** protection against progression from asymptomatic HIV infection to AIDS = 90%.

**Probability** of TB Diagnosis = 90%.

**BCG + Booster Vaccine Efficacy** = **30% relative to BCG alone.**

In “best” and “worst” case scenarios, key parameters were varied simultaneously: cost of booster vaccine, prevalence of initial single and multi-drug resistance, TB annual risk of infection, prevalence of HIV at birth, the probability of TB diagnosis, the cost of a DOTS visit, ART protective efficacy against HIV progression, and booster vaccine efficacy.

In the “best case” scenario, values for all these parameters were doubled, with the exception of the cost of booster vaccine, ART protection against HIV progression, and the probability of TB diagnosis, which were both halved, and the protective efficacy of combined vaccination compared to BCG alone, which was set to 70%. With the best case scenario, we predicted cost savings of $38.56 million, with prevention of 6,038 active TB cases and 4,854 TB-related deaths over 10 years.

In the “worst case” scenario, all key parameters were halved, except that the unit cost of the booster vaccine was doubled, the probability of TB diagnosis was set to 90%, ART reduced the probability of progression from asymptomatic HIV infection to AIDS by 90%, and the additional protective efficacy of combined vaccination compared to BCG alone was set to 40%. With this combination of assumptions, estimated costs were $7.32 million greater than for BCG alone, with 941 active TB cases and 137 TB-related deaths averted. A change in the proposed mechanism of vaccine action, with the booster assumed to protect against acquisition of *M. tuberculosis* infection, resulted in substantial cost savings, as well as further reductions in TB cases and related deaths (Table 7).

**Figure 4:**
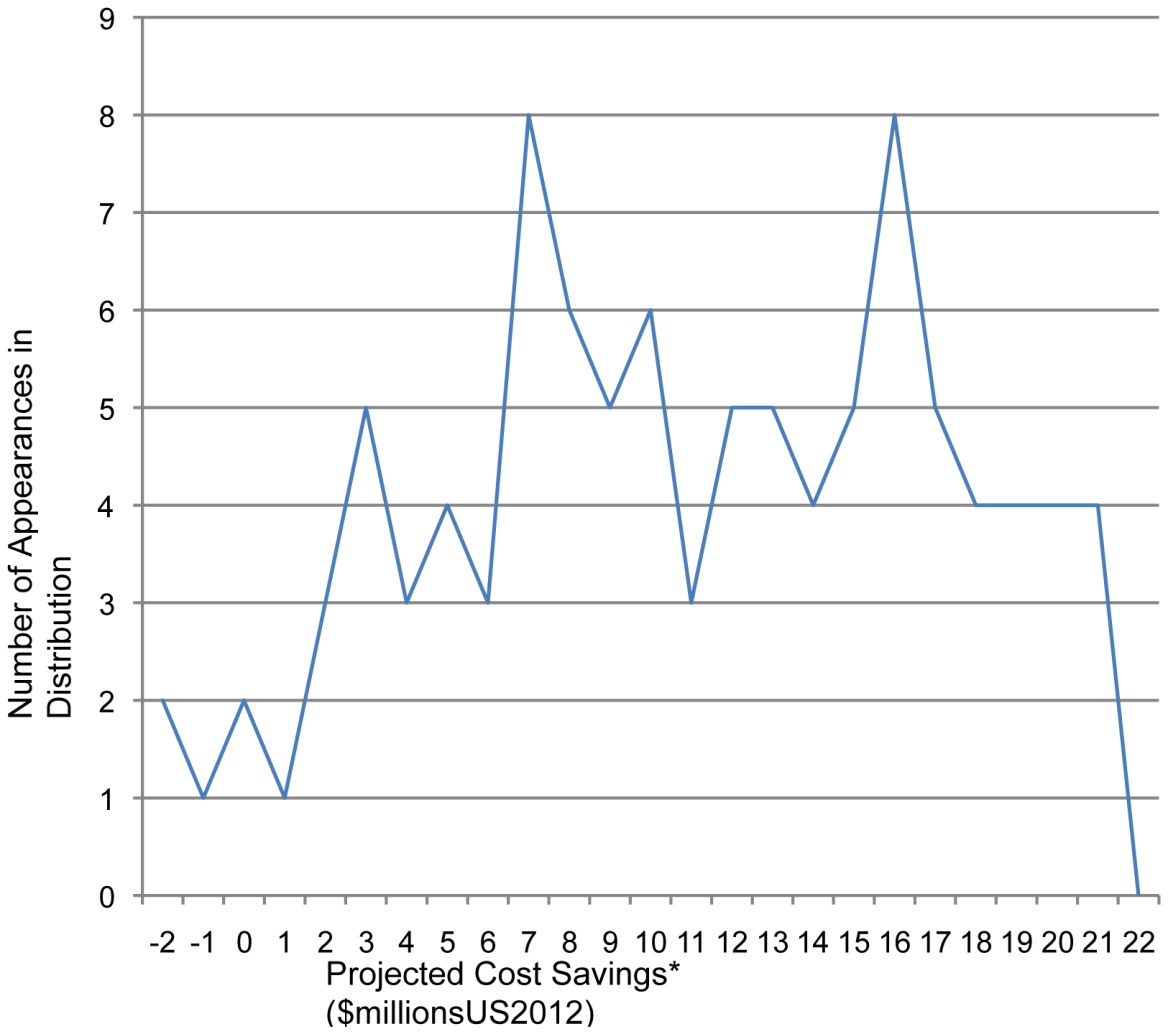
Monte Carlo distribution of projected cost savings using the Booster Vaccine Strategy. *Parameters varied include TB ARI, discount rate, the cost of lost work time for family members, HIV prevalence at birth, the total cost of a DOTS visit and booster vaccine primary efficacy.

In the probabilistic sensitivity analysis we varied six key parameters: annual risk of TB infection, discount rate, HIV prevalence at birth, DOTS program cost, cost of lost work time for family members, and the booster vaccine's efficacy. The most likely cost for the booster strategy was consistently lower than that for the BCG alone strategy, with mean associated cost savings of $11.21 million; median savings were $11.53 million, with interquartile range $6.3–16.8 million (Figure 4).

## Discussion

From a societal perspective, infant booster vaccination with a new tuberculosis vaccine appears less costly than BCG vaccine alone, if the new vaccine has at least modest protective efficacy - even after clinical trial costs and various unfavorable assumptions are considered. These results are concordant with previous analyses addressing the potential impact of a pre-exposure TB vaccine. Findings were generally similar in a previous analysis examining neonatal BCG replacement with or without a booster [27]. In that analysis, only at assumed protective efficacy values below the 40% lower limit used in the present analysis, was the replacement vaccine strategy more costly than BCG alone. In the recent infant Phase IIB study of MVA85A, there was no additional efficacy beyond BCG alone [11]. Clearly, without large-scale clinical trial data supporting a more effective candidate vaccine, any predictions of cost-effectiveness remain hypothetical. Nonetheless, extensive sensitivity analyses suggest that a modestly effective booster vaccine will likely prove cost-effective. However, if mass vaccination is to occur, pricing must be within reach of low and middle-income countries, which is unlikely to be the case with the high-end estimate ($15/dose) we used in sensitivity analysis.

Abu-Raddad and colleagues estimated that an improved neonatal vaccine could reduce TB incidence by 39%–55%. They further concluded that a catch-up vaccination campaign, using a vaccine that provides pre-exposure protection, could reduce incidence by as much as 67%, in the absence of HIV infection [28]. Another analysis suggested that a pre-exposure vaccine might be associated with a one-third drop in TB incidence, over the longer term [29].

A strength of the present analysis is that all costs were derived from previously published surveys, or interviews with OETC. Previous cost data for patients and families allowed us to adopt a societal perspective. Epidemiologic parameters were taken primarily from South African data. The remainder were taken from previously published models or statistics from similar settings. Our analysis is relevant to high TB incidence settings, including those with a high prevalence of HIV infection.

Our model assumed that major TB epidemiologic parameters remained constant over the course of the simulation, apart from vaccine effects. Hence we did not consider other emerging strategies to improve TB control, such as improved diagnostic capacity or expanded treatment of latent TB infection [30], [31]. Our analysis used a static Markov model; as it focused on childhood TB over a short time frame, we did not consider secondary transmission (which is rarely the result of TB in young children), or herd immunity by vaccination.

The ultimate costs of vaccine research and development remain uncertain. We were able to incorporate some initial estimates from OETC, and we prorated these costs to South Africa's global share of BCG vaccination. It is premature to accurately estimate research and development costs, and several very large and costly clinical trials may ultimately be required before any candidate vaccine is suitable for licensure and distribution. The extent to which these costs will ultimately be included in the commercial vaccine purchase price remains uncertain, as do eventual production costs and profit margins. However, our sensitivity analysis did address the potential impact of varying research, development, and production costs—and of varying profit margins–on the vaccine purchase price.

At present, the absence of validated biomarkers for protective immunity against *M. tuberculosis* infection and disease means that clinical vaccine trials must use active TB as their primary endpoint. Even in very high TB incidence settings, such as sub-Saharan Africa, this entails very large sample sizes, and the attendant costs. However, the present analysis suggests that from a societal standpoint, the necessary investments of resources, time and money are likely to pay off in terms of cost savings as well as improved health.
